# Isolation of Underivatized Amino Acids for Radiocarbon Analysis Using a Porous Graphite Column

**DOI:** 10.1021/acs.analchem.5c02922

**Published:** 2025-10-13

**Authors:** Christian Heusser, Lukas Wacker, Negar Haghipour, Timothy I. Eglinton, Thomas M. Blattmann

**Affiliations:** † Department of Earth Sciences, 27219 ETH Zürich, 8092 Zürich, Switzerland; ‡ Laboratory of Ion Beam Physics, 27219 ETH Zürich, 8093 Zürich, Switzerland; § PSI Paul Scherrer Institute, 5232 Villigen, Switzerland; ∥ Asian School of the Environment, Nanyang Technological University, Singapore 639798, Singapore

## Abstract

We developed a method for isolation and purification of individual underivatized amino acids for compound-specific radiocarbon analysis. Our method employs a semipreparative porous graphitic carbon column that allows for separation and isolation of all proteinogenic amino acids in a single step. To minimize contamination, we utilized a mixed-mode ion-exchange column to further purify the isolated amino acid fractions. Radiocarbon measurements of standard amino acids processed with this method resulted in F^14^C values closely aligned with their original F^14^C values. From these measurements, we calculated the blank contribution to 1.0 ± 0.3 μg C with F^14^C = 0.6 ± 0.12, rendering this approach suitable for ultrasmall samples with enhanced measurement reliability.

## Graphical abstract

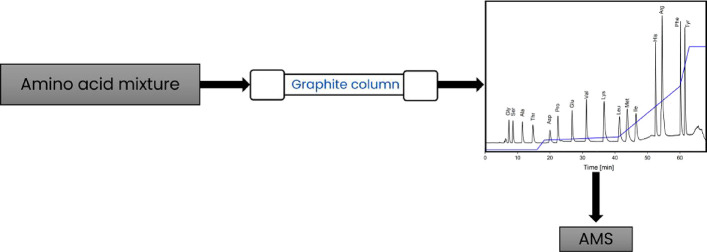

Compound-Specific Radiocarbon Analysis (CSRA) has emerged as a powerful tool in the field of analytical chemistry, offering unprecedented insights into the age and origin of individual compounds within complex mixtures.^1–3^ Among the various applications of CSRA, the analysis of amino acids holds significant promise due to their fundamental roles in biological processes, their diversity in chemical structures, and their presence in diverse environmental and archeological samples.^4^

Amino acids are ubiquitous organic molecules and play a crucial role in both the carbon (C) and nitrogen (N) cycles, acting as fundamental building blocks of proteins and serving as intermediates in metabolic pathways.^5^ Understanding their dynamics can provide valuable insights into biochemical pathways and ecological interactions. Accurate radiocarbon dating of amino acids can thus enhance our knowledge of these cycles and their influence on various environmental and biological systems.

The separation and purification of amino acids from complex environmental mixtures poses substantial challenges. Previous studies have explored various high-performance liquid chromatography (HPLC) methods for the separation and isolation of underivatized amino acids.^6–12^ Despite advancements, achieving baseline separation of individual amino acids, especially small amino acids (e.g., glycine, serine, alanine),^6,7,9^ remains a persistent challenge due to carbon contamination. Studies suggest that carbon contamination in isolated fractions often arises from column bleed, solvents, and the coelution of undesired compounds.^8,13,14^ These sources of contamination can interfere with radiocarbon measurements, underscoring the need for meticulous optimization of chromatographic conditions to ensure the integrity of isolated amino acids.

Recent advancements in Accelerator Mass Spectrometry (AMS) have significantly decreased the sample size required for radiocarbon measurements at natural abundance levels.^15,16^ Modern AMS instruments now routinely measure samples containing less than 20 μg C.^17,18^ This technological progress is particularly advantageous in fields where sample material is limited (e.g., Hendriks et al.^19^). However, the reduction in sample size also means that the potential impact of any extraneous carbon introduced during sample preparation is greatly amplified.^20^ It is thus crucial to develop methods with minimized carbon contamination during the isolation and purification processes. Previous studies have indicated that HPLC-isolated amino acids contain significant amounts of contamination of up to 5 μg C.^6–8^

Here, we present an advanced approach for the isolation of underivatized amino acids specifically tailored for CSRA. The method leverages advanced chromatographic techniques to achieve high-resolution separation of amino acids from complex matrices. By optimizing parameters such as column selection and mobile phase composition, we aim to improve the range, purity and yield of isolated amino acids, thereby enhancing the reliability and expanding the applicability of radiocarbon measurements.

## Experimental Section

### Standards and Solvents

Powdered standards of 14 amino acids, glycine (Gly), l-serine (Ser), l-alanine (Ala), l-threonine (Thr), l-aspartic acid (Asp), l-proline (Pro), l-glutamic acid (Glu), l-valine (Val), l-leucine (Leu), l-methionine (Met), l-isoleucine (Ile), l-histidine (His), l-arginine (Arg), and l-phenylalanine (Phe) were purchased from Sigma-Aldrich. Gly, Ala and Met are radiocarbon-dead, whereas the other amino acids are radiocarbon-modern. For each amino acid, a 0.1–0.2 M liquid standard was prepared in 0.1 M HCl. Aliquots of the amino acid liquid standards were combined to make a standard with 14 individual amino acids (concentrations of individual amino acids approximately 0.01 M) (AAmix) (Figure 1a). For calibration and testing of the HPLC system, a mixture of 17 amino acids (AAS18) (Figure 1b) was purchased from Sigma-Aldrich. LC/MS-grade acetonitrile and HPLC-grade dichloromethane (CH_2_Cl_2_) were purchased from Fisher Scientific AG, nonafluoropentanoic acid (NFPA) (>98%) was purchased from TCI Chemicals, and trifluoroacetic acid (TFA) (>99%, suitable for HPLC), ammonium hydroxide (NH_4_OH) (25%, for analysis) and hydrochloric acid (HCl) (37%, NORMAPUR) were purchased from VWR Chemicals, and Amberchrom 50W X8 (200–400 mesh) cation exchange resin, inhibitor-free diethyl ether (>99.9%) and hydrofluoric acid (HF) (38–40%, EMPLURA) were obtained from Sigma-Aldrich and were used in our procedures.

**Figure 1:**
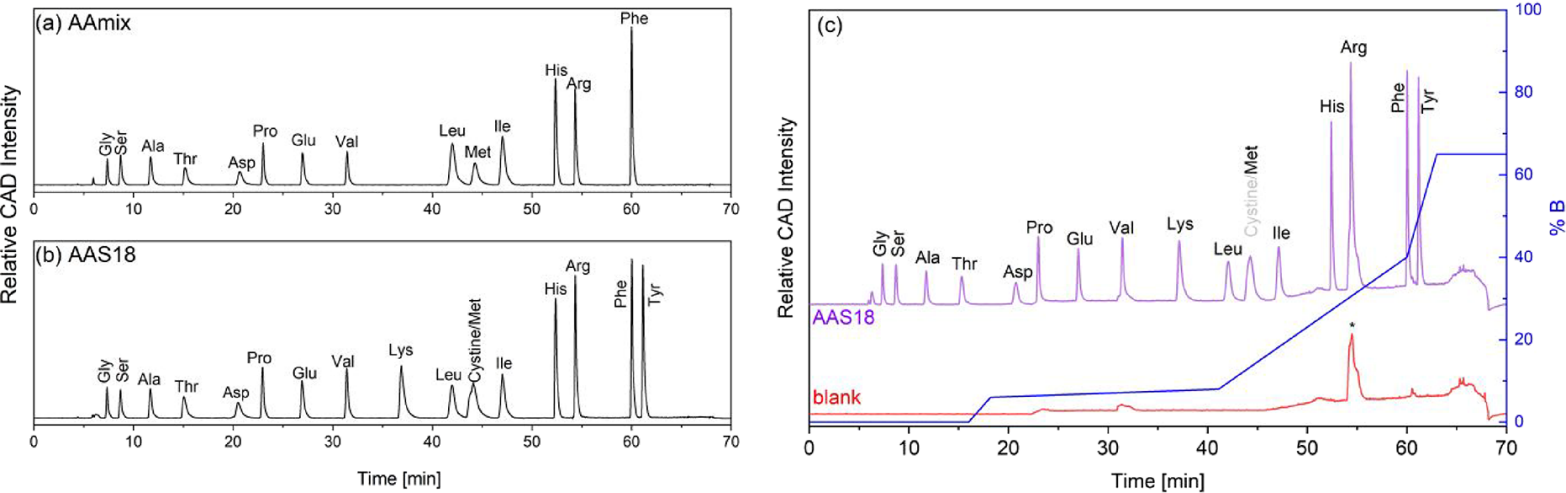
Background-subtracted HPLC chromatograms of (a) AAmix (10 μL injection volume), (b) AAS18 (50 μL injection volume), and (c) injections of AAS18 (purple, 10 μL injection) and blank (red). The asterisk (*) marks a landmark peak, that coincides with Arg. The gradient of the eluent as fraction of solvent B is shown in blue.

### HPLC System and Isolation of Amino Acids

Individual amino acids in the mixed solution were separated and isolated by HPLC (1260 series, Agilent Technologies, CA, USA). The HPLC system comprised an online degasser, a binary pump, an autosampler, a column thermostat, a diode-array detector (DAD), a fraction collector, and a third-party charged aerosol detector (Corona CAD, Thermo Fisher Scientific, MA, USA). The HPLC was equipped with a reversed-phase porous graphitic carbon (PGC) column (Hypercarb PREP, semipreparative scale, 10 × 250 mm, particle size 5 μm, Thermo Scientific, Runcorn, UK) in combination with a guard column (Hypercarb, 4 × 10 mm, particle size 5 μm, Thermo Scientific, Runcorn, UK) for the separation and isolation of the amino acids as shown in Figure 1c. For the purification of the isolated amino acid fractions, the HPLC was equipped with a mixed-mode ion-exchange reversed-phase column (Primesep A, semipreparative scale, 10 × 50 mm, particle size 5 μm, SIELC Technologies, IL, USA) in combination with a guard column (Primesep A, 4.6 mm, particle size 5 μm, SIELC Technologies, IL, USA). We used a precolumn filter (ColumnSaver, 0.5 μm, Supelco) to protect the columns from particulate material. Mobile phases were ultrapure water with 23 mM NFPA (Solvent A), pure acetonitrile (Solvent B), ultrapure water with 0.1% (v/v) TFA (Solvent C), and acetonitrile with 0.1% (v/v) TFA (Solvent D). NFPA and TFA were used as ion-pairing reagents and surface-active agents for the separation of underivatized amino acids by HPLC.^21^

For the PGC column, the column thermostat was set to 25 °C. The injection volume was set at 100 μL, and a total of 3 injections were performed. Prior these injections, the HPLC system was equilibrated by injection of 10 μL AAS18 standard and a blank injection. The solvent gradient was as follows: 0 to 16 min (A: 100%; B: 0%), to 18.2 min (A: 94%; B: 6%), to 41.1 min (A: 92%; B: 8%), to 60 min (A: 60%; B: 40%), to 63 min (A: 35%; B: 65%), to 70 min (A: 35%; B: 65%) at a constant flow rate of 3.5 mL/min. The column was then flushed with 100% B for 10 min at 5 mL/min followed by equilibration with 100% A for 30 min at 3.5 mL/min for cleaning and preconditioning for the next run.

For the mixed-mode column, the column thermostat was set to 20 °C. The injection volumes were set at 50 or 100 μL for each run. Here, amino acids were purified with a constant flow rate of 4 mL/min under different isocratic conditions. For Gly, Ser, Ala, Thr, Asp, Glu, and Pro the isocratic mixture consisted of 95% C and 5% D. For Val, the isocratic mixture was 85% C and 15% D. For Leu and Ile the isocratic mixture was 75% C and 25% D. For Phe, His and Arg, the isocratic mixture was 60% C and 40% D. After each run, the column was flushed with 100% D for 5 min at a flow rate of 5 mL/min, followed by an isocratic mixture of 60% C and 40% D at a flow rate of 4 mL/min for 5 min and equilibration with the desired isocratic mixture for 5 min at a flow rate of 4 mL/min. This program was chosen as it provided the lowest background signals as monitored on the CAD detector.

### Collection of Amino Acids

Prior to amino acid collection, the HPLC system was connected to a CAD to assess the retention time of each amino acid. For the Corona CAD, the N_2_ gas pressure was set at 60.5 ± 0.1 psi (corona voltage: <2.40 kV) and an evaporation temperature of 50 °C was set. Due to the high flow rate, an adjustable flow splitter (UP P-470, IDEX H&S, NY, USA) was used. We observed that the CAD signal lagged the DAD signals by approximately 1%. This discrepancy was attributed to the non-native integration of the CAD detector with the HPLC system, resulting in the CAD signal ending slightly earlier than the DAD signals. After peak identification, the flow line to the CAD was disconnected and then connected directly to the fraction collector. The amino acids were isolated using the fraction collector with a time-based trigger mode (Figure 2) (for collection windows, see Table S1). Potential drift of retention time was monitored by the DAD absorbance at 220 nm. The amino acids collected with the PGC column were dried and redissolved in 350 μL of 0.1 M HCl for subsequent injection into the mixed-mode column under conditions described above. The collected amino acids were dissolved in 350 μL of 0.1 M HCl and then injected into the mixed-mode column. For all amino acids, the injections were divided into one 50 μL injection and three 100 μL injections. Specifically, for Gly, Ser, and Ala, we collected the amino acids as follows: one 50 μL injection, one 100 μL injection, and two additional 100 μL injections (collected separately). For the remaining amino acids, we collected the 50 μL injection and combined the fractions of the three 100 μL injections.

**Figure 2:**
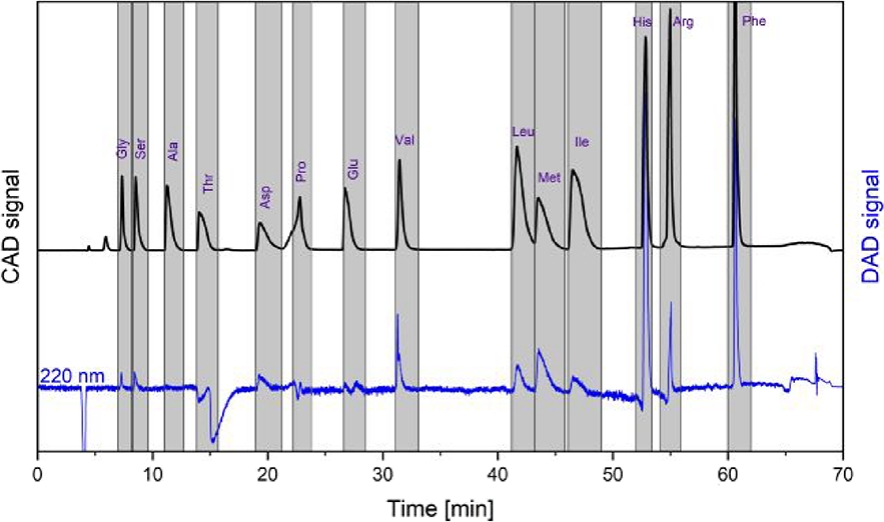
Fraction collection windows (gray) of amino acids. Background-subtracted CAD (black) and DAD (blue) chromatograms of AAmix are shown as reference. The injection volume was set at 100 μL. Fraction collection windows are also given in Table S1.

### Purification of Amino Acids

The collected fractions were transferred into preheated 20- or 40 mL glass vials (6 h at 450 °C), where the samples were dried under a constant stream of N_2_ gas at 70 °C. Following procedures recommended in Ishikawa et al.,^7^ the samples were redissolved in 0.5 mL of 0.1 M HCl and introduced into Eppendorf tubes with internal filters (wwPTFE NANOSEP MF, pore size: 0.2 μm, ODPTFE02C34, Pall Life Sciences, MI, USA) to remove precipitates and solid residues. Prior to use, the Eppendorf tubes were leached and centrifuged (Eppendorf Centrifuge 5418) at 14,000 rpm for 90 s three times with 0.5 mL of 0.1 M HCl. The filtered samples were transferred and dried in precombusted 0.9 mL V-bottom glass vials. The samples were dried on a hot plate at 70 °C under a constant stream of N_2_ gas. The dried samples were then washed twice with ∼100 μL of fresh, inhibitor-free diethyl ether to remove possible column bleed contamination.

### Validation of Purity

The isolated AA were dissolved in 100 μL of 0.1 M HCl. The purity of the amino acids was checked by injecting a small aliquot (0.5 μL) on an analytical-scale PGC column (Hypercarb, 2.1 × 150 mm, particle size 5 μm, Thermo Fisher Scientific), monitoring eluent with CAD. Samples that showed a single peak after the initial injection and monovalent cation peaks were deemed pure and prepared for radiocarbon analysis.

### HPLC Procedural Blanks

To assess the HPLC procedural carbon blanks derived from solvents, column bleed and other sources, we injected 100 μL of 0.1 M HCl twice into the HPLC system equipped with the PGC column. Fractions were collected in the same time frames as used for the AAmix. After the isolation with the PGC column, the collected fractions of the procedural blank were dried and redissolved in 200 μL of 0.1 M HCl. The fractions were then injected into the HPLC equipped with the mixed-mode column. The isocratic mixtures were chosen to be the same as the corresponding AA fractions.

### Case Study of a Soil Sample

We also hydrolyzed a Podzol soil sample following the method described by Blattmann et al.^6^ Briefly, we demineralized 5 g of freeze-dried soil with 100 mL HF in a closed 360 mL PFA jar (Savillex, MN, USA) at 60 °C. After 3 days, the lid was opened to dry the sample. After complete evaporation of the remaining HF, the sample was transferred into a PFA hydrolysis vessel (Savillex, MN, USA) and hydrolyzed in 6 M HCl at 115 °C for 16 h followed by drying in a rotary evaporation system. The hydrolysate was then desalted by loading it onto a column packed with Amberchrom 50W X8 (200–400 mesh) cation exchange resin followed by rinsing with water until pH became neutral, and the amino acids were liberated with 10% NH_4_OH. After drying, the sample was dissolved in 0.5 mL of solvent A and filtered to remove solid residues.

### Radiocarbon Analysis and Blank Calculations

Isolated amino acids and blanks were transferred into tin capsules (3.5 × 5.5 × 0.1 mm, 0.04 mL, Elementar, Germany). Prior to use, tin capsules were soaked in CH_2_Cl_2_ for 30 min and then dried. Tin capsules were placed on a Petri dish placed on a hot plate (120 °C) and sample solution was added by syringe. Upon reaching dryness, the capsule was then folded using tweezers and transferred to precombusted 1.5 mL glass vials for storage prior to analysis. Radiocarbon analysis was performed on an elemental analyzer (EA) coupled to a MICADAS (Mini Carbon Dating System) AMS system at the Laboratory of Ion Beam Physics, ETH Zürich.^15,22–24^

Data processing was done using the in-house BATS software.^25^ In all cases, radiocarbon measurements are reported as F^14^C, which is the ratio of the radiocarbon content in a sample to that of a modern reference standard based on atmospheric carbon dioxide levels in 1950. We applied the model of constant contamination (eq 1) for the evaluation of the mass and F^14^C of extraneous carbon and extraction of the F^14^C of samples (F^14^C_s_) from the AMS measurements (F^14^C_m_).^20^

$$F^{14}C_{s}=\frac{F^{14}C_{m}*m_{m}-F^{14}C_{c}*m_{c}}{m_{m}-m_{c}}$$ 1

where F^14^C_c_ is the contaminant F^14^C and *m*_m_ is the total measured C mass (measured by thermal conductivity detector on the EA), which is the sum of the actual C mass of the sample *m*_s_ and of the contamination *m*_c_.

The corresponding uncertainty of F^14^C_s_ is derived from error propagation (eq 2):

$$\sigma_{F^{14}C_{s}}^{2}={\left[\sigma_{m_{c}}\left(\frac{F^{14}C_{m}*m_{m}-F^{14}C_{c}*m_{c}}{(m_{m}-m_{c}{)}^{2}}\right)-\left(\frac{F^{14}C_{c}}{m_{m}-m_{c}}\right)\right]}^{2}+{\left[\sigma_{m_{m}}\left(\frac{F^{14}C_{m}}{m_{m}-m_{c}}-\frac{F^{14}C_{m}*m_{m}-F^{14}C_{c}*m_{c}}{(m_{m}-m_{c}{)}^{2}}\right)\right]}^{2}+{\left[\sigma_{F^{14}C_{m}}\frac{m_{m}}{m_{m}-m_{c}}\right]}^{2}+{\left[\sigma_{F^{14}C_{c}}\frac{-m_{c}}{m_{m}-m_{c}}\right]}^{2}$$ 2

The correction for constant contamination was done using Gly (F^14^C = 0.009) and Ser (F^14^C = 1.069).

## Results and Discussion

### Separation of Amino Acids

The isolation of individual amino acids for CSRA requires baseline chromatographic separation. Our analysis using the PGC column achieved successful baseline separation for 15 out of 17 amino acids in the AAS18 standard (Figure 1b). We found that the separation was maintained even for injection amounts of 800 to 1500 nmol per individual amino acid on column (Figure 2). For such large injections, we observed that the retention times of the amino acids were slightly shorter (Figures S1 and S2). However, the shorter retention times did not lead to coelution of the amino acids.

Methionine and cystine exhibited very similar retention times and coeluted as a single peak in the chromatogram. Given the relatively low natural abundance of these two amino acids,^26^ their incomplete separation was deemed acceptable and could be tackled in a separate step if needed.

During the analysis, a characteristic background peak was observed with the CAD detector between 53 and 55 min, coeluting with Arg (Figure 1c). This has been attributed to the gradient program employed rather than the presence of a carbon-containing component.^27^

As a case study, we applied the method to the desalted hydrolysate of a Podzol soil, that was prepared with the method described in Blattmann et al.^6^ As expected, the chromatogram of the soil (Figure 3) was more complex compared to the standard amino acid mixture. Prominent peaks between 5 and 7 min in the chromatogram of the soil hydrolysate show the presence of monovalent inorganic cations. Although the dominance of this peak indicated a high salt load, it did not alter the retention times of the amino acids. We also confirmed the presence of many amino acids in the soil hydrolysate. Interfering compounds were particularly prominent in the chromatogram between 50 and 57 min with several peaks, interfering with Arg. This highlights the importance of the mixed-mode column, which helps with purification by removing these interferences. We also note that we observed no peak for Met, indicating the low abundance of this amino acid in the soil sample.

**Figure 3:**
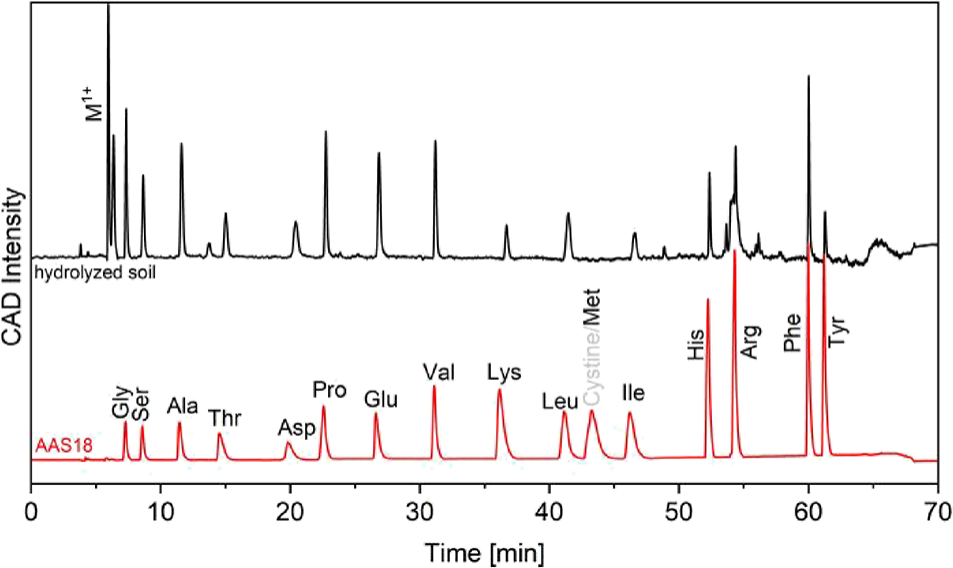
Background-subtracted chromatograms of a soil hydrolysate (top, black) and AAS18 (bottom, red). M^1+^ marks the elution of monovalent metals such as Na^+^, K^+^, or NH_4_^+^.

Despite achieving good baseline separation with the PGC column, the collected fractions from the first separation were further purified using an orthogonal separation chemistry with a mixed-mode column. This step was added to help with the further removal of coeluting matrix compounds and to aid in the elimination of NFPA (Figure S5). In the early stages of method development, improved radiocarbon results were observed by using both separation chemistries in sequence, compared to using only the PGC column. The use of a short mixed-mode column was appropriate for this task as the amino acid isolates from the PGC column were simply purified further in a chromatogram that regularly consisted of one peak, which additionally offered several advantages such as reduced run time and cost efficiency due to lower eluent consumption, and the fact that short columns are less expensive than long columns. Furthermore, by employing isocratic separation schemes in this second separation, greater comparability of blanks between different amino acids was made possible as several of them had identical aquatic-organic mobile phase ratios which also affect solvent residue and column bleed-resulted contamination. These benefits enhance both the efficiency and economy of the purification process as well as the data processing. The removal of coeluting compounds is an issue that is especially important from complex environmental matrices that limits the applicability of a single PGC column separation approach. In the case of biological matrices such as for targeting hydroxyproline from collagen,^28^ future method development may seek to optimize with using the PGC column only as coeluting uncharacterized compounds will unlikely be an issue and greater time efficiency and sample recovery will be advantageous.

Overall, the combination of the PGC and mixed-mode columns proved effective for the isolation and purification of amino acids, facilitating reliable CSRA. Further optimization of the gradient program could potentially mitigate the background peak observed, improving the overall resolution and accuracy of the analysis.

### Radiocarbon Blank

The procedural blank collected during the retention times relevant for the collection of the individual amino acids revealed no CAD-detectable extraneous compounds. However, all samples that underwent chromatographic isolation showed an additional signal peak in the subsequent mixed-mode column reflecting the presence of salt. The retention time of this salt peak was between the initial injection peak and before the signal of glycine, which is the first amino acid to elute and is aligning with the signals of monovalent cations.^27^ We concluded that these peaks do not include carbon and were derived from solvents and glassware. We followed the procedures for post-HPLC purification described by Ishikawa et al.^7^ to reduce carbon contamination. Overall, we found a constant contamination of 1.0 ± 0.3 μg C with F^14^C = 0.6 ± 0.12, which is in good agreement with Ishikawa et al.^7^

### Radiocarbon Analysis of Standard Amino Acids

We measured 11 out of standard amino acids purified with the procedures described above. Arg could not be purified due to a failure of the Primesep A column, while Asp and Met were measured using EA-AMS due to instrumental failure. The F^14^C results for the procedural amino acids (F^14^C_m_) were compared with the unprocessed amino acids (F^14^C_ref_) (Table 1). We found that the F^14^C_m_ of the ^14^C dead amino acids (Gly and Ala) were higher than their F^14^C_ref_. For the F^14^C_m_ of the ^14^C modern amino acids, we observed that smaller samples were generally lower than F^14^C_ref_, while larger samples were higher (Figure S3). However, using amino acids with alternating ^14^C content we found no carryover of neighboring amino acids.

**Table 1:** Results of Radiocarbon Measurements of Procedural Amino Acid Standards^a^

sample ETH lab code	amino acid	*m*_m_ (μg C)	F^14^C_ref_	F^14^C_m_	F^14^C_s_
139264.1.1	glycine	2	0.009	0.186 ± 0.012	<0.4
139264.1.1	glycine	5	0.009	0.165 ± 0.007	0.027 ± 0.065
139265.1.1	glycine	26	0.009	0.034 ± 0.002	0.007 ± 0.011
139268.1.1	alanine	13	0.010	0.061 ± 0.002	0.006 ± 0.018
139269.1.1	alanine	22	0.010	0.031 ± 0.002	<0.018
139270.1.1	alanine	51	0.010	0.031 ± 0.002	0.017 ± 0.005
139272.1.1	histidine	16	1.060	1.039 ± 0.009	1.063 ± 0.016
139273.1.1	histidine	28	1.060	1.034 ± 0.008	1.048 ± 0.011
139277.1.1	serine	12	1.069	1.025 ± 0.010	1.058 ± 0.020
139278.1.1	serine	25	1.069	1.058 ± 0.008	1.075 ± 0.012
139279.1.1	serine	56	1.069	1.073 ± 0.008	1.080 ± 0.009
139282.1.1	threonine	8	1.058	0.985 ± 0.014	1.030 ± 0.031
139283.1.1	threonine	64	1.058	1.054 ± 0.018	1.060 ± 0.019
139288.1.1	leucine	34	1.100	1.093 ± 0.009	1.106 ± 0.011
139289.1.1	leucine	227	1.100	1.106 ± 0.009	1.107 ± 0.009
139292.1.1	proline	12	1.089	1.064 ± 0.010	1.100 ± 0.021
139293.1.1	proline	73	1.089	1.100 ± 0.009	1.106 ± 0.009
139296.1.1	glutamic acid	14	1.075	1.052 ± 0.010	1.082 ± 0.018
139297.1.1	glutamic acid	89	1.075	1.084 ± 0.008	1.088 ± 0.009
139300.1.1	isoleucine	28	1.088	1.083 ± 0.009	1.099 ± 0.012
139301.1.1	isoleucine		1.088	1.070 ± 0.010	1.075 ± 0.010
139304.1.1	valine	49	1.078	1.031 ± 0.010	1.038 ± 0.010
139308.1.1	phenylalanine	40	1.105	1.095 ± 0.009	1.106 ± 0.010
139309.1.1	phenylalaine	230	1.105	1.100 ± 0.009	1.102 ± 0.009

a The uncertainties reported here are ± 2σ. More details on F^14^C_ref_ values are given in Table S2.

The biggest difference between F^14^C_m_ and F^14^C_ref_ was found for Gly (ETH lab code: 139264.1.1 and 139265.1.1). These samples were very small (2 and 5 μg C, respectively), and therefore carried a large proportion of contamination. This was also observed for one Thr sample (ETH lab code: 139282.1.1) with a sample size of 8 μg C. The deviation of F^14^C_m_ from F^14^C_ref_ decreases with increasing sample size. One notable exception is Val (ETH lab code: 139304.1.1), that shows a relatively large deviation of F^14^C_m_ from F^14^C_ref_ despite a relatively large sample size (49 μg C).

After applying corrections to account for constant contamination, the F^14^C_s_ for 23 out of 24 samples (10 out of 11 amino acids) fell into 2σ range of the F^14^C_ref_ values (Figure 4). This is partly due to the degree of contamination, the uncertainty of the C masses especially for very small samples, and the impurity contained in the original standard materials. We also found that the F^14^C_s_ of Ser, Leu, Pro, Glu and Ile were more modern than their F^14^C_ref_, which we attributed to impurities contained in the original standard materials that were removed during chromatography. One sample, Val, fell out of the 2σ range and had a significantly lower F^14^C_s_ value than F^14^C_ref_. This is partly because of the degree of contamination and ^14^C enriched impurities in the standard material, suggesting that further investigation is warranted to fully understand the underlying causes. However, this is beyond the scope of the present work.

**Figure 4:**
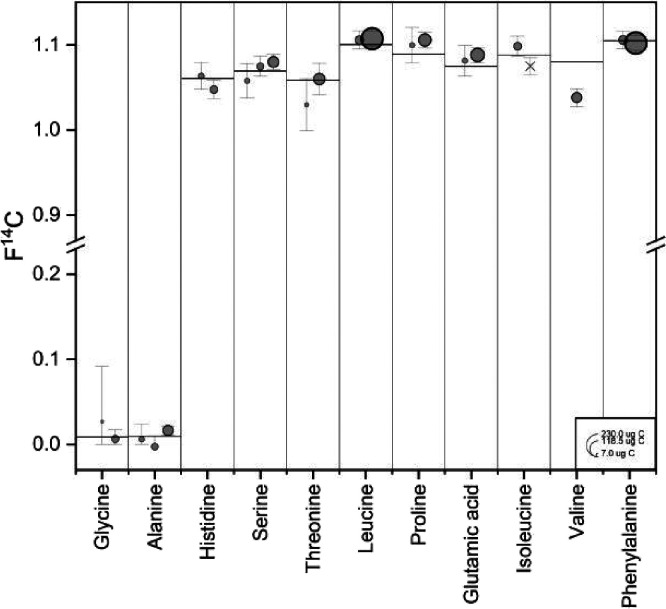
Blank-corrected F^14^C_s_ values of HPLC processed amino acids. The horizontal lines represent F^14^C_ref_ values of the individual amino acid standards. HPLC-processed samples are shown as dots. Dot sizes represent the individual sample sizes. The uncertainties reported here are ± 2σ.

### Column Performance

While most HPLC columns are packed with silica with modified surface groups, the column used in our work is packed with porous graphitic carbon. Therefore, this column has different advantages over silica-based columns. Most importantly, the absence of modified surface groups makes PGC columns very robust with minimal column bleed.^29^ Additionally, PGC columns can be operated over the entire pH range, is compatible with all solvent systems and can also be used for routine high-temperature chromatographic applications with temperatures of up to 200 °C.^30^ We also observed that re-equilibration was relatively short (30–40 min), thus increasing the throughput.

We further investigated the reproducibility of our method and the robustness of the column. We found that the PGC column achieved reproducible chromatograms over more than 140 injections for 6 months including soil hydrolysate samples (Figure 5). We observed that the retention times of Asp, Pro, Lys, Leu, Met, and Ile had slight variations. This was attributed to variations in the concentrations of NFPA. For fraction collection, we found that it is therefore important to occasionally insert runs with a standard mixture to adjust for possible shifts in retention times.

**Figure 5:**
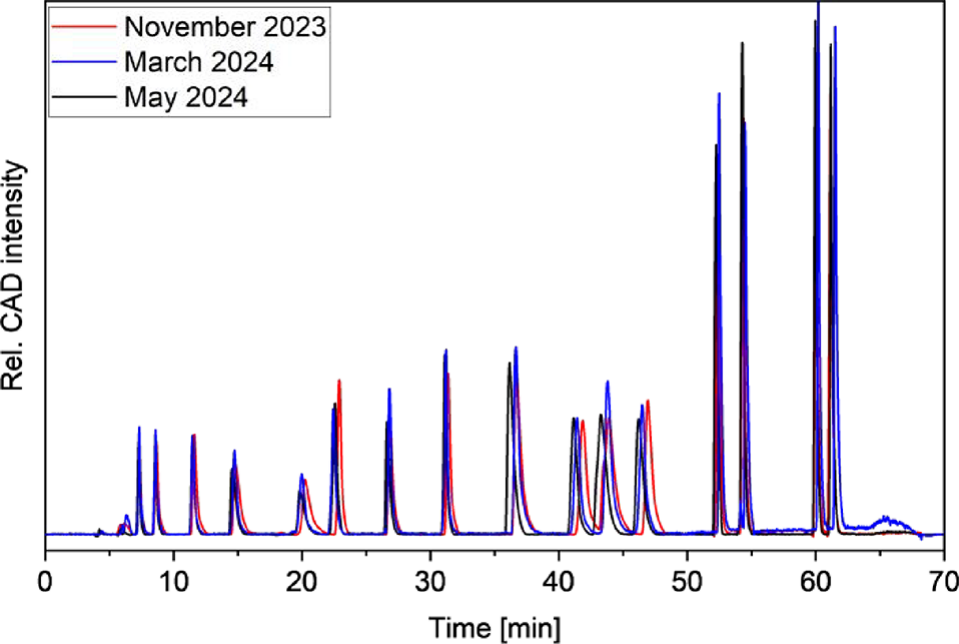
Column Performance over the course of 6 months and approximately 140 injections. The chromatograms shown here are from November 2023 (red), March 2024 (blue), and May 2024 (black). The injection volumes of the shown chromatograms were 10 μL each.

For the mixed-mode column, we observed increases of the background signal as well as retention time shifts after about 100 injections. This wear is most likely caused by the degradation of the modified surface groups.

## Conclusions

We have developed a method that allows the rapid isolation of (multi)­microgram quantities of underivatized amino acids for CSRA. Our method allows separation of most proteinogenic amino acids in a single step. A second chromatography is then used for further purification of the amino acids.

We found a total of 1.0 ± 0.3 μg C of contamination, which is an improvement over existing methods (Bour et al.:^8^ 1.8 μg C for early eluting amino acids and 4.5 μg C for late eluting amino acids; Ishikawa et al.:^7^ 1.5 ± 0.2 μg C).

We conclude that our method is suitable for ^14^C analysis on ultrasmall samples (down to 15 μg C). The ability to isolate most proteinogenic amino acids in a single step makes it suitable for future applications in archeology and biogeochemistry.

## Supplementary Material



## Notes

Fraction collection windows and 14C analysis of reference amino acid standards and Additional chromatograms and uncorrected F14C data (PDF)
