# Supplementary material for: Isolation of Underivatized Amino Acids for Radiocarbon Analysis Using a Porous Graphite Column
Source: Anal Chem. 2025 Oct 13;97(42):23150–6. doi: 10.1021/acs.analchem.5c02922 (PMC12573229; doi:10.1021/acs.analchem.5c02922)
Supplement: Supplementary file 1 [file ac5c02922_si_001.pdf]

Isolation of Underivatized Amino Acids for Radiocarbon Analysis Using a Porous Graphite Column

Christian Heusser,*[♠♣♪] Lukas Wacker,[♣] Negar Haghipour,[♠♣] Timothy I. Eglinton,[♠] and Thomas M. Blattmann*[♠ஃ]

[♠]Department of Earth Sciences, ETH Zürich, 8092 Zürich, Switzerland

[♣]Laboratory of Ion Beam Physics, ETH Zürich, 8093 Zürich, Switzerland

[♪]PSI Paul Scherrer Institut, 5232 Villigen, Switzerland

[ஃ]Asian School of the Environment, Nanyang Technological University, 639798 Singapore

*Corresponding author: Christian Heusser christian.heusser@psi.ch and Thomas M. Blattmann thomas.blattmann@ntu.edu.sg

Table of Content

- Table S1: Fraction Collection Windows for Amino Acids
- Table S2: ^{14}C analysis of reference amino acids standards.
- Fig. S1: Chromatograms of different injection amounts of AAS18 standard.
- Fig. S1: Blank-subtracted chromatograms of different injection amounts of AAS18 standard.
- Fig. S2: Uncorrected $F^{14}C_m$ values of HPLC processed amino acids.
- Fig. S4: Representative chromatograms of amino acids separated with a Primesep A column.

Table S1: Fraction Collection Windows for Amino Acids

Amino Acid	Fraction Collection Window (min)
Gly	7.0–8.2
Ser	8.3–9.6
Ala	11.0–12.7
Thr	13.8–15.7
Asp	18.9–21.2
Pro	22.2–23.8
Glu	26.6–28.5
Val	31.1–33.1
Leu	41.2–43.2
Met	43.2–45.8
Ile	46.1–49.0
His	52.0–53.4
Arg	54.1–55.9
Phe	60.0–62.0

Table S2: ^{14}C analysis of reference amino acids standards. The uncertainties reported here are $\pm$ 2σ.

ETH lab code	Amino Acid	$F^{14}C_{ref}$
84863.1.1	*Gly*	0.009±0.001
144096.1.1	*Ser*	1.069±0.001
84855.1.1	*Ala*	0.010±0.001
84873.1.1	*Thr*	1.058±0.010
144098.1.1	*Pro*	1.089±0.001
144099.1.1	*Glu*	1.075±0.001
71369.1.1	*Val*	1.078±0.012
144097.1.1	*Leu*	1.100±0.001
144100.1.1	*Ile*	1.088±0.001
144095.1.1	*His*	1.061±0.001
84870.1.1	*Phe*	1.105±0.010

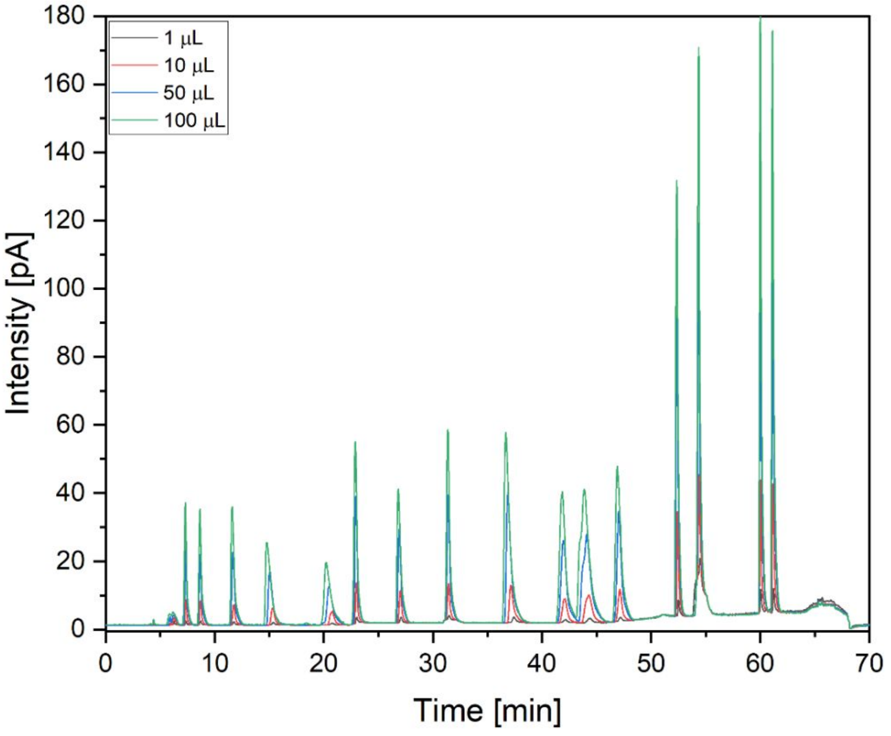

Fig. S3: Chromatograms of different injection amounts of AAS18 standard. Individual amino acid concentrations are 2.5 mM (1.25 mM for cystine), corresponding to 2.5 nmol/μL.

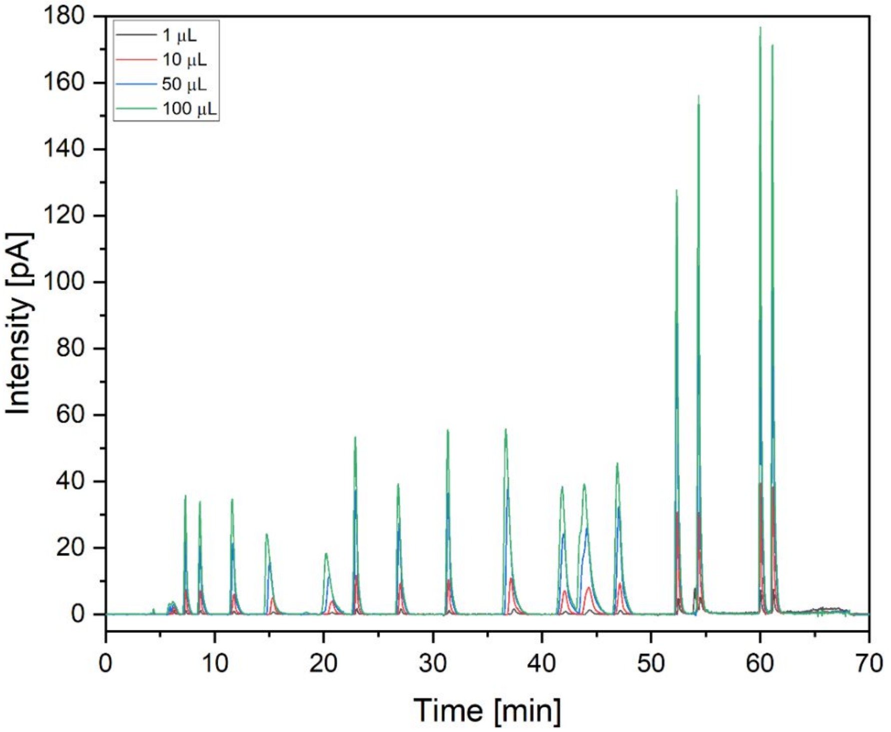

Fig. S4: Blank-subtracted chromatograms of different injection amounts of AAS18 standard. Individual amino acid concentrations are 2.5 mM (1.25 mM for cystine), corresponding to 2.5 nmol/µL.

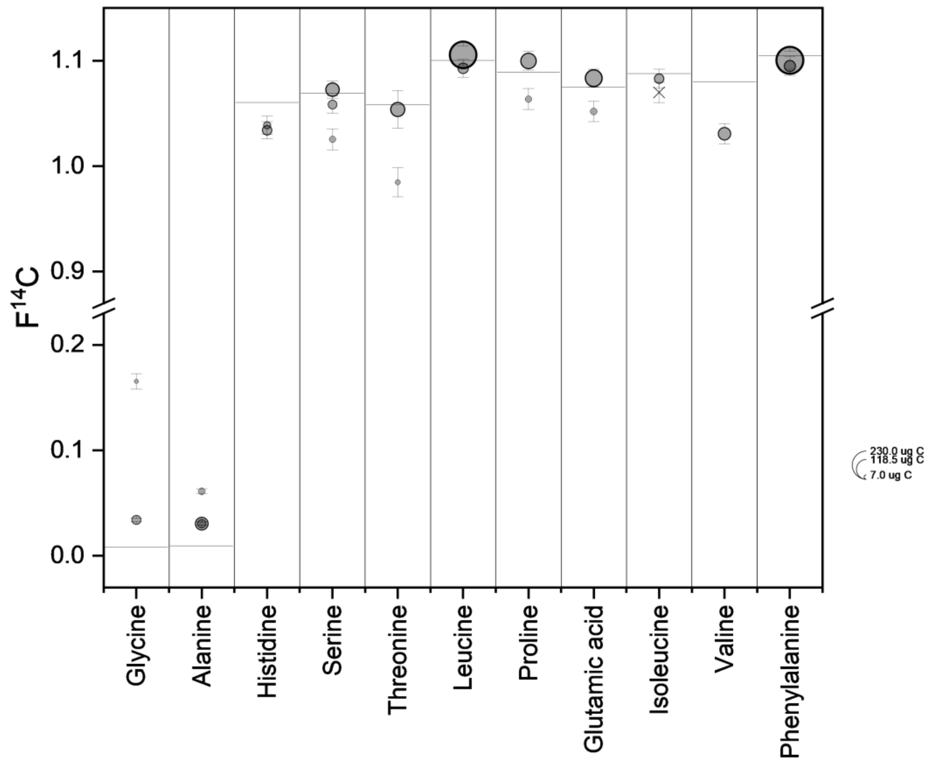

Fig. S5: Uncorrected $F^{14}C_m$ values of HPLC processed amino acids. The horizontal lines represent $F^{14}C_{ref}$ values of the individual amino acid standards. HPLC-processed samples are shown as dots. Dot sizes represent the individual sample sizes. The uncertainties reported here are ± 2σ.

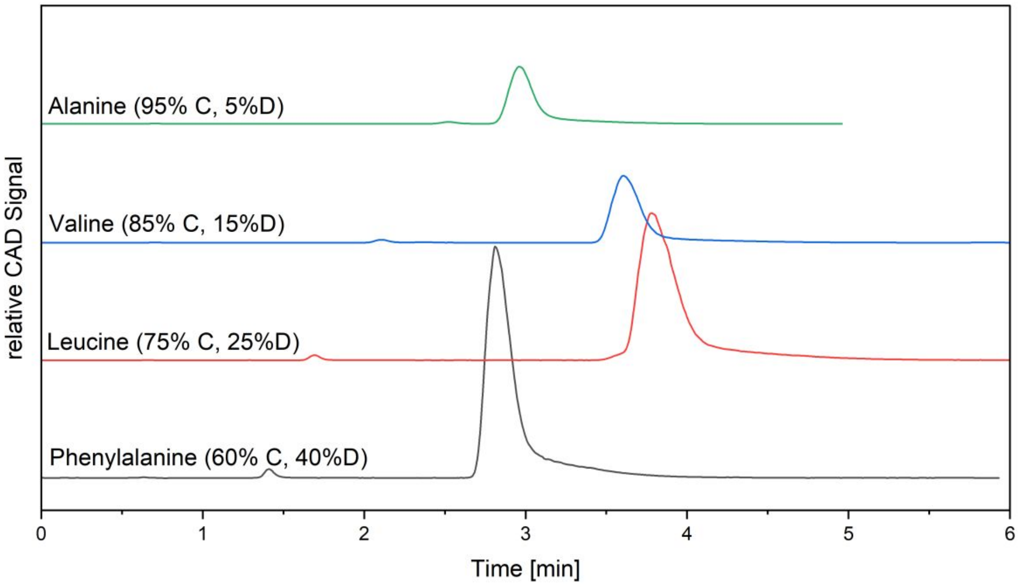

Fig. S4: Representative chromatograms of amino acids alanine (green), valine (blue), leucine (red) and phenylalanine (black) that were separated with a Primesep A column. For alanine, the program ended at 5 min because reconditioning starts.

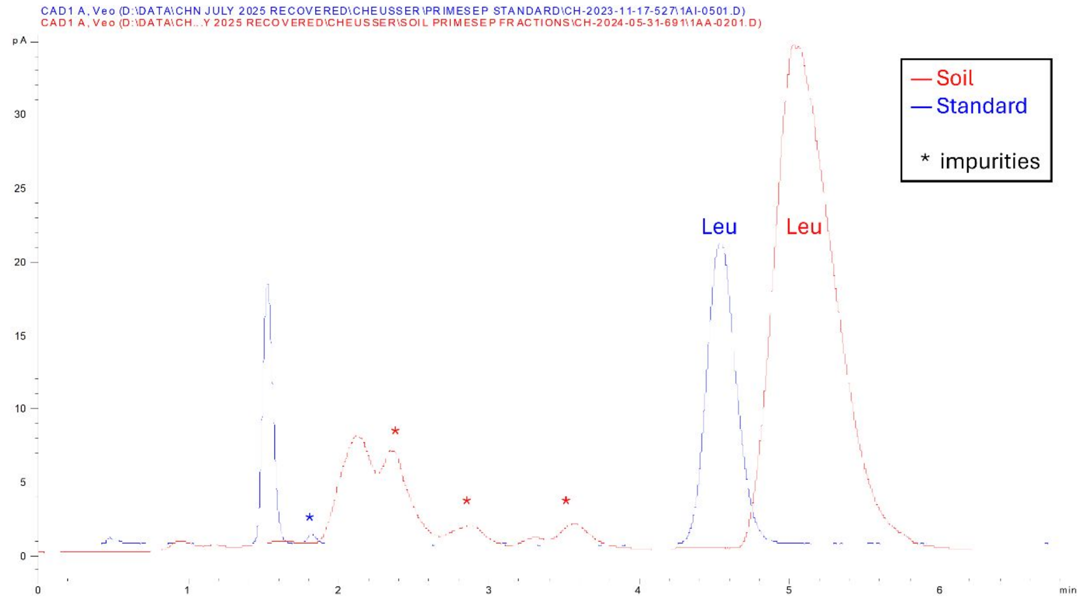

Fig. S5: Qualitative comparison of Primesep A chromatograms with CAD detector of leucine standard (blue) and from a soil sample (red) previously processed through PGC column. Several additional peaks besides leucine are clearly visible, indicating the presence of co-eluting impurities. This is a common observation on both CAD and UV signals.
